# The Effect of Topical Local Anesthetics on Thermal Pain Sensitivity in Patients with Irritable Bowel Syndrome

**DOI:** 10.1155/2012/438674

**Published:** 2012-02-28

**Authors:** Anthony Rodrigues, Christopher D. King, Fong Wong, Joseph L. Riley, Siegfried Schmidt, Andre P. Mauderli

**Affiliations:** ^1^Department of Child Neurology, Floating Hospital for Children at Tufts Medical Center and Tufts University School of Medicine, Boston, MA 021111, USA; ^2^Department of Behavioral Sciences, College of Dentistry, University of Florida, Gainesville, FL 32610, USA; ^3^Department of Restorative Dental Sciences, College of Dentistry, University of Florida, Gainesville, FL 32610, USA; ^4^Community Health and Family Medicine, College of Medicine, University of Florida, Gainesville, FL 32610, USA

## Abstract

Generalized hypersensitivity that extends into somatic areas is common in patients with irritable bowel syndrome (IBS). The sensitized state, particularly assessed by experimental methods, is known to persist even during remissions of clinical pain. It was hypothesized that disease-related nociceptive activity in the gut maintains a systemic-sensitized state. The present study evaluated responses to prolonged thermal stimuli maintained at constant temperature or constant pain intensity during stimulation. The effect of topically applied rectal lidocaine on heat sensitivity was also evaluated. The question is whether silencing potential intestinal neural activity (which may not always lead to a conscious pain experience) with lidocaine attenuates sensitization of somatic areas. Tests were also performed where lidocaine was applied orally to control for systemic or placebo effects of the drug. The IBS subjects exhibited a greater sensitivity to somatic heat stimuli compared to controls; however, lidocaine had no discernible effect on sensitization in this sample of IBS patients, where most of the individuals did not have clinical pain on the day of testing.

## 1. Introduction

Irritable bowel syndrome (IBS) is a common gastrointestinal disorder that consists of abdominal pain that can be associated with abnormal bowel movements [1]. The pain often fluctuates and can go into remission [2]. Since natural variation of visceral pain occurs in IBS and other visceral pain disorders, one method to evaluate altered pain processing in IBS is to psychophysically assess responses to controlled experimentally induced stimuli [3, 4]. Like other chronic pain cohorts, IBS patients are often more sensitive to a range of experimentally applied stimuli. For example, distensions of the rectum with a balloon in IBS patients are often reported as more painful and produce pain at lower thresholds compared to individuals without IBS [5–8].

While the underlying pathophysiological mechanism(s) are still unclear, increased sensitivity to visceral pain in IBS patients appears to be influenced by altered processing of afferent information from the gut [7, 8]. In a recent review by Zhou and Verne [3], several models suggest that IBS is associated with physiological changes in gastrointestinal tissue, which usually follow some type of injury or inflammatory insult (e.g., Salmonella infections). One proposed pathway to enhanced pain processing includes changes to intestinal permeability [9, 10] and low-grade inflammation [11], which in turn produces an upegulation of pronociception factors (e.g., splice variants of N-methyl-D-aspartic acid receptors, NMDA [12, 13]). Altered activity within primary visceral afferents secondarily induces changes in central processing mechanisms (e.g., NMDA-receptor-based signaling [14]), which manifests itself as allodynia and hyperalgesia to stimuli within the affected gastrointestinal tissue [3]. Thus, augmented nociceptive input from gastrointestinal tissue leads to increased visceral sensitivity and, consequently, clinical symptoms.

It has been argued that changes in the neural activity of the gut can have consequences beyond the symptomatic region and lead to sensitization of somatic regions. Recent studies have shown that IBS-related changes are not limited to the gut but expand to somatotopically remote regions such as the neck and head [15]. IBS patients are usually more sensitive to experimental stimuli applied not only to dermatomes corresponding to the gut but also to remote areas that do not share the same viscersomatic convergence [5, 8, 15–19]. Functional imaging studies have supported the idea of altered central processing of painful visceral and somatic pain in IBS patients (for review [20]). The relationship between visceral and somatic sensitivity in IBS patients is intriguing since it is possible that aberrant nociceptive signals from the symptomatic visceral tissues induce somatic hypersensitivity across the body [21]. If so, temporary interruption of nociceptive signals from the gut should allow this hypersensitivity to decline. The idea of a regional nociceptive focus is supported by recent studies in IBS and myofascial pain syndrome where local anesthetics were used in an attempt to silence a nociceptive focus that was presumed to maintain a generalized sensitized state [22–24]. Both have demonstrated a reduction in pain hypersensitivity in remote areas following anesthetic intervention, which provides support for the idea that a regional nociceptive focus (i.e., gut, muscle) plays a key role in the maintenance of hypersensitivity. However, other reports have put this view into question: topical rectally administered lidocaine failed to reduce pain sensitivity [25] and “trigger point” injections with saline or simply an empty needle were no less effective in reducing pain in myofascial pain patients than lidocaine injections [26]. The previously mentioned IBS studies also show the impact of factors other than the pharmacological effect of lidocaine, including placebo, in driving the changes in sensitivity. The disagreement between studies clearly suggests that questions remain regarding the mechanism that maintains widespread somatic sensitization in regional pain disorders.

The present study was designed to address two objectives. The first objective was to confirm and extend the observation that IBS patients were more sensitive to prolonged thermal stimuli even on days when clinical pain was in remission and that sensitization can extend to distant somatic regions (i.e., the upper extremities). For the first trial, a recently described stimulation methodology [27, 28] was used, which maintains a predetermined average pain intensity by automatically adjusting the temperature of a Peltier-based thermode as a function of the subject's rating on a visual analog scale (i.e., temperature decreased when ratings go above the set point). In this test (the first in the series) the subjects were unable to identify the treatment condition based upon how the experimental stimulus was perceived because the average pain intensity remained the same, regardless of the treatment condition. A second test used a conventional fixed stimulus temperature protocol. It was expected that IBS patients, when compared to normal controls, would exhibit somatic thermal hypersensitivity in both protocols. The second objective was to determine the effects of rectal lidocaine on somatic hypersensitivity. While designed to replicate previous research in IBS patients [22–24], the experimental design also controlled for placebo effects including the application of lidocaine in the oral cavity, which is not affected by the symptoms of IBS. It was expected that topical rectal but not oral lidocaine would lead to a reduction in somatic sensitization.

## 2. Methods

### 2.1. Subjects

Participants of the study were recruited from diverse social settings and had no close contact with each other. Recruitment and study procedures were approved by the University of Florida Institutional Review Board. Written informed consent was obtained from all participants. The criteria for membership in the control group required (a) no significant spontaneous pain anywhere in the body, (b) no ongoing pharmacotherapy with narcotics or antidepressants, (c) no disease or condition that might significantly affect pain perception or unduly increase risk of injury (e.g., neurological disorders, serious psychiatric disorders, diabetes, hypertension, serious cardiovascular disorders, pregnancy, and chronic pain diseases such as fibromyalgia syndrome), (d) no prior complications or allergies with the local anesthetic lidocaine. The criteria for the disease group required a diagnosis of ongoing IBS based upon the Rome II criteria [29], supplemented by additional criteria: absence of other diseases (including other chronic pain diseases), risk factors, and ongoing drug treatments, as described for the control group. Rome II criteria [24] are twelve weeks or more in the past 12 months of abdominal discomfort or pain that has two out of three features: relieved with defecation; onset associated with a change in frequency of stool; and onset associated with a change in form of stool. Patients with a history of widespread pain (e.g., individuals with fibromyalgia syndrome) were excluded from the study. Initial screening consisted of blood pressure measurement, completion of a health questionnaire, and a clinical diagnosis for IBS patients by a physician. Subject recruitment did not control for menstrual phase or contraceptives.

### 2.2. Testing Methodology

#### 2.2.1. Questionnaires

 The subjects were given three psychological assessment forms during the training session (session 1) to evaluate overall levels of depression (Beck Depression Inventory [30]), anxiety (State Anxiety Trait Inventory [31]), and somatization (Symptom Checklist [32]).

#### 2.2.2. Pain Measurement

Spontaneous clinical pain and experimental pain (induced by thermode) were measured with an electronic version of a visual analog scale [33] as mentioned previously [17, 27]. The electronic visual analog scale (eVAS) consisted of a low-friction sliding potentiometer of 100 mm travel. The left endpoint of the scale was identified as “no pain,” while the right endpoint was labeled as “intolerably intense pain.” The position of the slider was electronically converted into a pain rating between 0 and 100%. The slider automatically returned to the left “no pain” position when so required by the protocol.

#### 2.2.3. Thermal Nociceptive Stimulation

Thermal stimuli were administered with a flat copper contact thermode (25 × 25 mm). The thermode was electronically held at the desired temperature by a Peltier thermoelectric device and was brought into light skin contact of reproducible force by solenoid activation.

### 2.3. Testing Protocol

Test sessions consisted of daily testing sessions across seven nonconsecutive days (Table 1). Subjects were scheduled irrespective of whether or not IBS patients had an acute exacerbation of symptoms on the day of testing. Following assessment of clinical pain (Section 2.3.1), pain sensitivity was measured with two different testing protocols. The first protocol (pain intensity clamping) used a prolonged thermal contact with automatically adjusted temperature that maintained a predetermined pain intensity rating (Section 2.3.2). The second protocol administered series of thermal pulses of fixed temperature and measured the pain intensity elicited by these pulses (Section 2.3.3). Subjects were not informed about the order and expected effects of the treatments in order to minimize rating bias. During three of the seven daily test sessions (sessions 2, 5, and 7), pain sensitivity was assessed in the absence of any lidocaine or vehicle treatment. An overview of the drug and placebo schedule is shown in Table 1. The double-placebo treatment, which consisted of both an oral and rectal administration of KY Jelly without an anesthetic, was always first (session 3). Lidocaine (Section 2.4) was applied rectally (session 4) and orally (session 6). On sessions when lidocaine was used rectally, KY Jelly was applied orally and vice versa. Lidocaine sessions were always followed by a testing session without lidocaine application to monitor for potential residual effects of the anesthetic that might contaminate the subsequent lidocaine session (sessions 5 and 7).

#### 2.3.1. Ratings of Clinical Pain

All participants began each daily session by rating their spontaneous pain of the upper (head, neck, shoulder, upper back, arms, and hands) and lower (low back, bowel, legs, and feet) parts of the body on the eVAS. The subjects were then asked to rate the unpleasantness of the single most intense clinical pain. Several of the patients reported spontaneous pain or discomfort during some of the sessions. For the control group, all these ratings were required to be below 5% (on the 0–100% eVAS scale).

#### 2.3.2. Prolonged Painful Stimulus at a Fixed Pain Intensity Rating (Trial 1)

Pain testing began with a prolonged thermal stimulus to the thenar eminence of the right hand (Table 2). As reported previously [27, 28], the stimulus was controlled as a function of the pain intensity rating and maintained 25% average pain intensity for 120 seconds by modulating the thermode temperature (dependent variable). Prolonged stimulation consisted of two phases: induction and maintenance phase. At the beginning of stimulation, the thermode temperature was 34°C, which did not elicit pain in any of the subjects. Then, during the induction phase, the temperature was slowly stepped up in 0.6°C/sec increments until a pain intensity of 10% was reached. At this point, the temperature continued to increase in 0.3°C increments. The induction phase ended and the maintenance phase began when the pain intensity rating first reached the set point of 25% on the eVAS. Temperature and eVAS rating were sampled once per second.

#### 2.3.3. Prolonged Painful Stimulus at Fixed Temperature (Trial 2)

The subject was repositioned to test the left thenar eminence. Pain sensitivity was assessed with a continuous thermal stimulus at a standardized temperature (Table 2). The thermode was held in contact with the skin while the temperature was ramped from 34 to 47 deg C (rate of 1.5 deg/sec) and then held at this temperature for 30 sec. Pain intensity was continuously rated on the eVAS during the stimulus.

### 2.4. Lidocaine

 For the lidocaine conditions, a jelly containing 2% lidocaine (150–300 mg per application; AstraZeneca) or a similar volume of placebo (KY Jelly) was digitally applied rectally and orally in all IBS and control subjects prior to testing on three of the seven daily testing sessions (Table 2). Pain testing began within 15 minutes after administration, when the lidocaine effect was expected to peak [34].

### 2.5. Data Analysis

 One-way ANOVA was used to test for group differences on measures of anxiety, depression, and somatization. Further relationships between psychological variables and psychophysical pain measures were determined with Pearson correlations. Repeated measures analysis of variance (ANOVA) was used for statistical analysis with age entered as a covariate. Bartlett's test of sphericity of the residual covariance matrix was used to test the sphericity assumption. Greenhouse-Geisser adjustments were made where appropriate. For trial 1, the factors were Group (IBS patient, healthy controls) and Treatment (average baseline; double placebo (session 3), rectal lidocaine (session 4), oral lidocaine (session 6)). One-way ANOVA was used to evaluate differences among the three baseline sessions (i.e., sessions 2, 5, and 7), which were averaged. The dependent variables were thermode temperature when pain rating first reached 10%, temperature at the end of the induction phase (i.e., when pain rating first reached set point of 25%), and the average temperature needed to maintain the set pain intensity throughout the window from 51–60 sec of the prolonged fixed painful stimulus. For trial 2, the factors for the fixed prolonged painful stimulus were Group and Treatment. The dependent variables were the second of the trial where the eVAS rating first reached 10%, peak pain rating during the trial, and the average pain rating across the trial.

## 3. Results

### 3.1. Clinical Characteristics of Subjects

Characteristics of the participants in the current study are presented in Table 3. An equal number of diarrhea-predominant female IBS patients (*n* = 11) between the ages of 18 and 52 years old and healthy controls (*n* = 11) between the ages of 20–54 completed the study. All subjects were right-handed.

### 3.2. Baseline Assessments of Psychological Profile

Scores of Symptom Checklist (i.e., somatization measure) were higher in IBS patients compared to healthy controls (Control: 96.8 ± 17.9; IBS: 102.7 ± 18.1; *F* (1, 21) = 5.129, *P* = 0.03). The groups did not differ on degree of depression (Control: 4.7 ± 3.6; IBS: 7.6 ± 6.4; *F* (1, 21) = 0.615, *P* > 0.10) or anxiety (Control: 29.9 ± 4.7; IBS: 38.6 ± 12.3; *F* (1, 21) = 0.615, *P* > 0.10). The psychological scores did not correlate with any psychophysical pain measures or treatment effects (All *P* > 0.10).

### 3.3. Group Differences to a Prolonged Painful Stimulus at a Fixed Pain Intensity Rating (Trial 1)

**Figure 1:**
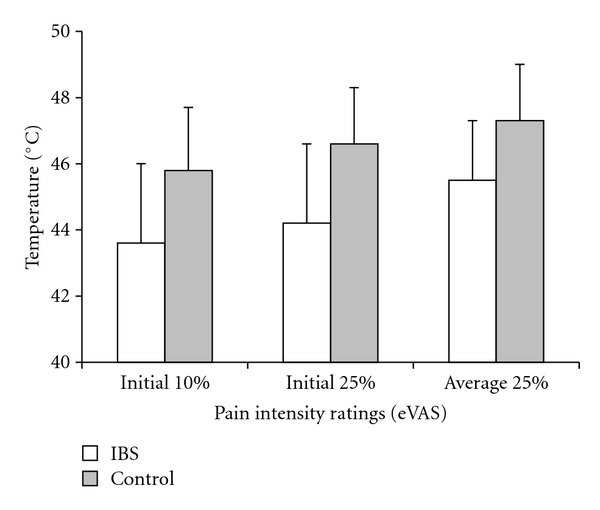
Continuous thermal stimulus applied to thenar eminence of right hand of IBS (open bars) and control (gray bars) participants during baseline sessions. Thermode temperatures during induction phase at the time when pain intensity first reached 10% and 25% were lower for the IBS group compared to controls. The average temperature needed to maintain pain intensity at 25% was also lower for the IBS group.

Differences across the 3 control sessions were tested for the three dependent variables: temperature of the thermode at an initial pain intensity rating of 10% and 25% (i.e., induction phase) and the average temperature to maintain a pain intensity rating of 25% (i.e., maintenance phase). The results of repeated measure ANOVA (Group × Day × Measure) indicated that there were no differences across the 3 control sessions (All *P* > 0.10). Consequently, data for the 3 control sessions were collapsed for all further analysis (nonsignificant main effect of session or interaction term involving session). However, differences were observed between IBS and control groups regarding measures during the induction and maintenance phase of thermal stimulation. In Figure 1, IBS patients as a group, when compared to their healthy counterparts, were significantly more sensitive to thermal nociceptive stimulation (i.e., the temperatures needed for eliciting any given pain intensity level were lower).

Table 4 shows the average temperatures during the induction and maintenance phases under different treatment conditions with lidocaine and placebo. Under all treatment conditions, a similar pattern was observed in which the IBS group needed lower temperatures to elicit the defined amount pain intensity during both phases. The data further suggest that the IBS and control groups were affected equally by the treatments, and that the effect of rectal and oral lidocaine application did not differ to a statistically significant degree. This finding does not support the notion that generalized sensitization of IBS patients in somatic areas distant from the gut (i.e., the hands) is primarily maintained by a localized nociceptive focus. For the initial pain rating of 10%, repeated measure ANOVA indicated a significant main effect of group (*F* (1,20) = 6.579, *P* = 0.018). The main effect of treatment (*F* (3,18) = 1.238, *P* > 0.05) and treatment × group (*F*(3,18) = 0.649, *P* > 0.05) interactions were not significant. For the initial pain rating at 25%, repeated measure ANOVA indicated a significant main effect of group (*F* (1,20) = 6.923, *P* = 0.016). However, the main effect of treatment (*F* (3,18) = 0.480, *P* > 0.05) and treatment × group (*F* (3,18) = 1.556, *P* > 0.05) interaction were not significant. Continuing this trend, the average thermode temperature to maintain a rating of 25% was lower in the IBS group. A repeated measure ANOVA indicated a significant main effect of group (*F* (1,20) = 5.731, *P* = 0.027), but the main effect of treatment (*F* (3,18) = 0.007, *P* > 0.05) and treatment × group (*F* (3,18) = 0.664, *P* > 0.05) interaction were not significant.

### 3.4. Group Differences to a Prolonged Painful Stimulus at Fixed Temperature (Trial 2)

Responses to a 30 sec 47°C stimulus are presented in Table 5. For the control sessions (session 2, 5, and 7), differences across the 3 control sessions were tested for the three dependent variables: time to pain intensity of 10%, peak pain intensity, and average pain intensity. The results of repeated measure ANOVA (Group × Session × Measure) indicated that there were no differences across the 3 control sessions, consequently, data for the 3 control sessions (sessions 2, 5, and 7 in Table 1) were collapsed for all further analysis.

As reported in Table 4, in Trial 1, the IBS group reached a pain intensity rating of 10% in a significantly shorter amount of time and their pain intensity ratings were higher compared to controls. However, these measures were not significantly affected by the treatment conditions. For the time to 10%, a repeated measure ANOVA indicated a significant main effect of group (*F* (1,20) = 5.380, *P* = 0.031). The main effect of treatment (*F* (3,18) = 0.604, *P* > 0.05) and treatment × group (*F* (3,18) = 0.1383, *P* > 0.05) interaction were not significant. In addition, a repeated measure ANOVA for peak pain intensity indicated a nonsignificant main effect of group (*F* (1,20) = 3.675, *P* = 0.070). The main effects of treatment (*F* (3,60) = 3.521, *P* = 0.036) and treatment × group (*F* (3,18) = 1.109, *P* > 0.05) interaction were significant. Finally, a repeated measure ANOVA for the average pain intensity indicated a significant main effect of group (*F* (1,20) = 4.933, *P* = 0.038). The main effects of treatment (*F* (3,60) = 1.260, *P* > 0.05) and treatment × group (*F* (3,18) = 1.579, *P* > 0.05) interaction were not significant.

## 4. Discussion

The goal of this study was to verify the existence of somatic hypersensitivity on glabrous skin of the hands in IBS patients and determine the interaction between potential intestinal neural activity and somatic sensitization. The current study confirmed the presence of somatic hypersensitivity, even during remissions of clinical pain and in a region distant from the segmental level of the gut (i.e., the hands). The sensitization was demonstrated with both protocols used in this study, that is, with the method that measured the temperature required to maintain a predetermined level of pain and the method that used a fixed stimulus temperature and measured the pain intensity elicited by it. The degree of somatic sensitization of the hands was not attenuated by rectal lidocaine, nor was it affected by topical lidocaine applied at the control location, the oral cavity.

Previous studies of our laboratory [17, 28, 35] and others [5, 8, 15, 16, 18, 19, 36] have supported the idea that IBS patients are more sensitive than healthy controls to a range of laboratory stimuli, including to heat. The data of the present study are in agreement with these earlier findings as the temperature needed to elicit a moderate pain intensity level of 25 on a scale of 0–100 tended to be approximately 2 degrees lower for the IBS group compared to the healthy control group. Likewise, IBS patients, when compared to healthy controls, rated the pain intensity elicited by a thermal pulse of given temperature as more painful.

A continuous barrage of tonic impulses emanating from symptomatic viscera and viscerosomatic convergence had been proposed as the basis for the induction and maintenance of sensitization to regions beyond the gut [5, 8, 15, 16, 18, 19, 36]. Some earlier studies [8, 15, 18, 23] that used thermal water immersion stimuli and threshold/tolerance measures found a declining sensitization gradient from the symptomatic segments to more proximal segments. Other experiments that used focal thermal cutaneous stimuli with a defined contact area to the cheek, forearm, and calf confirmed the existence of widespread cutaneous sensitization but failed to demonstrate a gradient effect [17]. Spinal sensitization of IBS patients was also demonstrated in a model of NMDA receptor-dependent temporal summation [36]. Most studies are in agreement in one finding: the central nervous system in IBS patients is altered beyond the symptomatic region of the gut.

The application of rectal topical local anesthetics offers a pharmacological tool to evaluate the contribution of neural activity within the gut to changes in neural processing that extend into distant somatic areas. This basic concept has already found support in experiments where pharmacological blockade of nociceptive input normalized clinical and stimulus-induced pain [37–39].

Our results are in agreement with two studies that examined visceral sensitivity following rectal lidocaine in normal subjects [25, 40] but may compliment earlier studies that were conducted in IBS patients [23, 24]. A number of factors and mechanisms need to be considered when analyzing the difference in results between studies.

1. *Placebo Effect*. Unlike the mentioned earlier studies, the present investigation included tests where topical lidocaine was applied orally to control for placebo and systemic effects. The subject was kept in the dark as to whether the oral or rectal application represented the “active” treatment. This was important because a subject that can identify lidocaine by its numbing effect in the area of application (i.e., anal region) may become biased and develop an uncontrolled placebo effect. Furthermore, the present study took into account the possibility that subjects could develop a reinforced placebo effect based upon how they perceive the experimental stimulus early on in the experimental series. If subjects realize that the stimuli appear to be less painful under a certain test condition, they may be biased later on in the experiment and perceive subsequent tests even less painful. The first test in the present study always used a pain intensity clamping method where the elicited pain intensity level was the same regardless of test condition. The temperature that was required to elicit that predetermined pain level (and that served as the response variable) was unknown to the subject. In the present study, a lidocaine effect on pain sensitivity was not detected and therefore concerns regarding a positive placebo effect are moot.
2. *Systemic Effect*. An earlier study [23] reported to have measured lidocaine blood levels to rule out systemic effects. It is well accepted that systemic lidocaine can lead to significant reductions in pain at very low blood levels (i.e., as low as 1 microgram/milliliter concentration) [41–44]. Systemic lidocaine is known to have an attenuating effect on pain responses in neuropathic conditions of human and animal models, as well as in human postoperative abdominal pain [45, 46]. Interestingly, intravenous lidocaine has been shown to reduce secondary mechanical hyperalgesia without affecting sensitivity thresholds [47]. It, therefore, cannot be ruled out that levels of lidocaine below the detection threshold of common blood assays might have an attenuating effect on pain. This raises the question whether the blood assay has been sensitive enough to conclusively rule out systemic effects of lidocaine on pain processing. Considering the fact that it may be difficult to reliably rule out any potential systemic lidocaine effects with blood tests, the present study's approach was to control for systemic effects with a control experiment where the drug was applied in a mucosa lined region distant from the gut (i.e., the oral cavity). A systemic lidocaine effect should have reduced pain sensitivity regardless whether lidocaine was administered rectally or intraorally. Systemic effects do not appear to be an issue in the present study because no lidocaine effect was detected in either location of application.
3. *Method and Location of Stimulation*. Somatic sensitization of Verne's et al. [23] and the present study was measured with different stimuli and in a different location. Verne et al. applied a water immersion stimulus to the foot, while the present study used a focal contact stimulus to the glabrous skin of the hand. One could argue that the lidocaine is able to affect somatic sensitization in the same segmental region as the gut (the foot) but be ineffective in regions that are segmentally distant, and therefore, enhanced pain sensitivity in IBS patients may rely on mechanisms other than “vicious cycle” type sensitization by viscerosomatic convergence.
4. *Patient Sample*. In the study by Verne and colleagues, the intensity of clinical (spontaneous) pain was at least 30 (on a scale from 0–100) during the experimental session while most subjects in the present study had no spontaneous pain on the days of testing. The present study effectively standardized the subject groups based upon their somatic pain sensitivity (all subjects in the IBS group were sensitized on the hand). Additional standardization based upon their clinical pain on the days of testing would have been advantageous. Standardizing subjects of the IBS group based upon their clinical pain would only be useful if it is assured that they have clinical pain of approximately the *same* intensity on *each* day of testing. This is more difficult to accomplish in a study with many sessions due to extensive controls for placebo and systemic effects.

Differences in symptom severity on the days of testing are a likely explanation for the difference in results between Verne's et al. study [23] and the present results. The hypothesis that rectal lidocaine can only reduce the portion of sensitization that is maintained by intestinal neural activity that is strong enough to result in a conscious pain experience needs to be considered. The portion of somatic sensitization that remains during remissions of clinical pain may no longer be reversible by the drug. This notion is consistent with a report [37] where neuropathic patients with greater pain severity exhibited greater analgesic response. Furthermore, in a followup to the aforementioned study, it was found that the qualitative experiences related to clinical pain determined the efficacy of lidocaine to reduce pain [39]. A factor analysis of pain qualities, which were collected from the short-form McGill Pain Questionnaire, revealed that a subgroup of patients with higher levels of “heavy” pain qualities experienced greater pain reduction following intravenous lidocaine. In this light, it may be understandable why in our study somatic sensitization was unresponsive to rectal lidocaine: most subjects' clinical pain was in remission on the day of testing. It is possible that somatic pain sensitization is not mainly induced or maintained by a nociceptive focus in the gut through a “vicious cycle” mechanism but a result of other factors (some of which may predate the clinical onset of IBS) including a deficit in tonic inhibition or descending facilitation. Events that occurred much earlier in life, such as stress, inflammation or dysfunctional endocrine functioning [48–56], could have induced plastic changes in the pain processing system and led to permanent widespread hyperalgesia. Indeed, symptom-free control subjects are occasionally encountered that are as sensitive in thermal pain tests as the most sensitive IBS patients [16]. It will be important in future studies to collect a thorough disease and trauma history from birth to the present from these individuals because it may provide clues regarding the cause of their sensitization. Once a widespread sensitized state is established, it will take only a small local insult to start a regional (e.g., IBS, MPS) or generalized (FMS) pain condition. In other words, preexisting changes in the pain processing system may have rendered these patients pain prone long before the spontaneous symptoms that define these pain diseases emerged. This argument finds support in animal models [57]. It has been possible to induce lifelong visceral and somatic hyperalgesia in rats with visceral insults early in life [58]. In these models, sensitization appears to persist even after visceral pathology associated with the insult is no longer visible. The issue could be put to rest by conducting a longitudinal study on large samples of symptom-free subjects. The predisposition theory would be supported if hyperalgesia can be demonstrated in a subpopulation of healthy subjects and that these individuals have a higher likelihood of developing pain conditions later in life [59]. The most clinically pressing question would then be how sensitization from an early life insult can be prevented from becoming permanent.

In conclusion, our results confirm that IBS patients are somatically sensitized even in regions that are segmentally distant from the gut. Earlier findings that somatic sensitization can be reduced by the application of rectal lidocaine are not confirmed by the present study. The most likely explanations for the different finding are that the effect of lidocaine (a) may not extend to somatic areas that are segmentally distant from the gut or (b) to sensitization that persists even during periods of symptom remission. Therefore, it can be argued that somatic sensitization that is maintained by nociceptive signals in the gut may be sensitive to lidocaine while another type of sensitization exists that is stable and no longer reversible by lidocaine. The present study points toward the existence of this latter mechanism without allowing detailed insights into its function.

## Figures and Tables

**Table 1:** Testing and treatment conditions over 7 nonconsecutive sessions. The seven sessions were spread out over two weeks, typically with 4 sessions during the first and 3 sessions during the second week. Following a training session, baseline sessions were assessed on sessions 2, 5, and 7. The fifth and seventh sessions were used to assess carryover effects of the treatment conditions. Treatment sessions were carried out on sessions 3, 4, and 6, which included a combination of rectally and orally administered anesthetic (i.e., lidocaine) and placebo. The order of anesthetic and placebo were not random. The anesthetic was administered either rectally (session 4) or orally (session 6) while the other site was given a placebo (i.e., oral placebo on session 4, rectal placebo on session 6).

Testing Conditions	Treatment Conditions	
Session	Rectal	Oral
(1) Training	No treatment	No treatment
(2) Baseline 1	No treatment	No treatment
(3) Treatment 1	Placebo (PL)	Placebo (PL)
(4) Treatment 2	Lidocaine (LID)	Placebo (PL)
(5) Baseline 2	No treatment	No treatment
(6) Treatment 3	Placebo (PL)	Lidocaine (LID)
(7) Baseline 3	No treatment	No treatment

**Table 2:** Order of experimental trials during each testing session. All trials were conducted during each session.

Trial	Stimulus Induced Pain	Testing site	Parameters
1	Prolonged thermal stimulus at fixed pain intensity	Right thenar	Temperature adjusted to maintain a pain rating ~25% (eVAS)
2	Prolonged thermal stimulus at fixed temperature	Left thenar	Pain ratings to a 30 sec pulse at 47°C

**Table 3:** Average clinical pain reports (±SD) across the treatment conditions in IBS subjects. The majority of IBS patients did report clinical pain at one time or another throughout the time period of their involvement in the study, but not always on the days of testing. None of the control subjects had any pain, and therefore control subjects are not listed in the table. No correlation was found between lidocaine effect and clinical symptoms during or between sessions in the lower or upper part of the body (All *P* > 0.10).

	Baseline (Session 1)	Double placebo (Session 3)	Rectal lidocaine (Session 4)	Oral lidocaine (Session 6)
Upper body intensity (0–100%)	22.0 (7.1)	22.0 (7.1)	19.0^†^	41.5 (13.4)
Lower body intensity (0–100%)	24.7 (14.2)	24.7 (14.2)	68.0^†^	23 (0.0)
Unpleasantness of clinical pain (0–100%)	37.3 (17.82)	37.3 (17.82)	82.0^†^	48.0 (2.83)
Number subjects reporting pain (%)	4 (36%)	4 (36%)	1 (9%)	2 (18%)

Abbreviations: ^†^, ratings for single subject.

**Table 4:** Average thermode temperatures (±SD) of prolonged painful stimulus at a fixed intensity rating between IBS and control participants (Trial 1). The baseline session represents data collapsed across sessions 2, 5, and 7. Participants rated a continuous thermal stimulus to induce 10% and 25% pain intensities during the induction phase and sustain 25% pain intensity during the maintenance phase for the various testing sessions with (rectal: *rectal lidocaine + oral placebo*; oral: *rectal placebo + oral lidocaine*) and without (placebo: *rectal placebo + oral placebo*) application of lidocaine.

	IBS				CONTROL			
	Baseline	Placebo	Rectal	Oral	Baseline	Placebo	Rectal	Oral
Induction								
10% (°C)	43.6 (2.4)	44.2 (2.1)	43.9 (2.7)	44.7 (2.7)	45.8 (1.9)	46.5 (2.2)	46.3 (1.8)	46.6 (1.2)
25% (°C)	44.2 (2.4)	44.1 (2.4)	46.3 (1.8)	44.2 (2.8)	46.6 (1.7)	46.6 (2.5)	46.4 (1.9)	46.8 (1.4)
Maintenance								
25% (°C)	45.5 (1.8)	44.8 (2.3)	45.8 (1.7)	45.8 (2.0)	47.3 (1.7)	47.0 (1.9)	47.3 (2.2)	47.7 (1.7)

**Table 5:** Average time to a pain rating of “10%” and eVAS ratings (±SD) of a 30 second pulse of 47.0°C on the left thenar (Trial 2). The baseline session represents data collapsed across sessions 2, 5, and 7. Pain intensity was continuously rated on a 0 to 100 scale. Time to obtain a rating of “10%” is measured in seconds. Continuous ratings of thermal pain were analyzed to determine peak (highest pain rating during the trial) and average (i.e., average amount of pain reported during the trial) pain.

	IBS				CONTROL			
	Baseline	Placebo	Rectal	Oral	Baseline	Placebo	Rectal	Oral
Time to 10% (sec)	4.7 (1.6)	4.5 (1.4)	4.2 (1.3)	4.6 (1.0)	7.8 (4.0)	6.1 (3.2)	6.4 (2.4)	6.4 (3.6)
Peak Pain	69.2 (21.4)	65.9 (32.3)	71.1 (24.7)	63.4 (22.9)	46.1 (20.7)	46.9 (20.0)	50.4 (23.9)	51.4 (23.6)
Average pain	41.5 (14.0)	44.6 (27.7)	44.3 (17.8)	39.0 (15.6)	24.3 (13.4)	22.7 (17.1)	28.3 (17.8)	29.5 (17.2)
